# Rodent models of neuroinflammation for Alzheimer’s disease

**DOI:** 10.1186/s12974-015-0291-y

**Published:** 2015-04-17

**Authors:** Amir Nazem, Roman Sankowski, Michael Bacher, Yousef Al-Abed

**Affiliations:** Elmezzi Graduate School of Molecular Medicine, The Feinstein Institute for Medical Research, 350 Community drive, Manhasset, NY 11030 USA; Institute of Immunology, Philipps University Marburg, Hans-Meerwein-Str., 35043 Marburg, Germany; Center for Molecular Innovation, The Feinstein Institute for Medical Research, 350 Community drive, Manhasset, NY 11030 USA

**Keywords:** Alzheimer’s disease, Neuroinflammation, Neurodegeneration, Animal models, Innate immunity, Amyloid-β, Tau protein

## Abstract

Alzheimer’s disease remains incurable, and the failures of current disease-modifying strategies for Alzheimer’s disease could be attributed to a lack of *in vivo* models that recapitulate the underlying etiology of late-onset Alzheimer’s disease. The etiology of late-onset Alzheimer’s disease is not based on mutations related to amyloid-β (Aβ) or tau production which are currently the basis of *in vivo* models of Alzheimer’s disease. It has recently been suggested that mechanisms like chronic neuroinflammation may occur prior to amyloid-β and tau pathologies in late-onset Alzheimer’s disease. The aim of this study is to analyze the characteristics of rodent models of neuroinflammation in late-onset Alzheimer’s disease. Our search criteria were based on characteristics of an idealistic disease model that should recapitulate causes, symptoms, and lesions in a chronological order similar to the actual disease. Therefore, a model based on the inflammation hypothesis of late-onset Alzheimer’s disease should include the following features: (i) primary chronic neuroinflammation, (ii) manifestations of memory and cognitive impairment, and (iii) late development of tau and Aβ pathologies. The following models fit the pre-defined criteria: lipopolysaccharide- and PolyI:C-induced models of immune challenge; streptozotocin-, okadaic acid-, and colchicine neurotoxin-induced neuroinflammation models, as well as interleukin-1β, anti-nerve growth factor and p25 transgenic models. Among these models, streptozotocin, PolyI:C-induced, and p25 neuroinflammation models are compatible with the inflammation hypothesis of Alzheimer’s disease.

## Introduction

After decades of research, Alzheimer’s disease (AD) remains incurable and thus is considered a major human healthcare challenge [1]. A therapeutic intervention with the potential to cure AD should be a mechanistic disease-modifying agent that can slow or halt the neurodegenerative process; and ideally reverse it towards regeneration [2]. Recently, several potentially disease-modifying agents have been suggested for AD. Many of these suggested therapeutic agents have passed the efficacy testing in animal models. However, all of the ensuing phase three clinical trials have failed [3]. These failures question not only our accurate understanding of the disease [1], based on which the therapeutic agents have been designed, but also the animal models in which both our understanding of the disease and drug discovery efforts are rooted [4].

Failure in AD drug discovery may in part be attributable to the so-called lesion seduction [1], a simplistic paradigm postulating that AD-related histopathological lesions are a direct reflection of its etiology [1]. Following this paradigm, the most commonly used animal models of AD are designed to recapitulate the lesions of AD [5], namely amyloid-β (Aβ) plaques and neurofibrillary tangles through transgenic induction of mutations related to amyloid and tau production (amyloid precursor protein (APP), presenilin-1 (PS1) and PS2, or tau mutations [6,1,7]). However, since late-onset AD (LOAD) is not caused by such mutations [6], the results from these animal models cannot be reliably extrapolated to the human condition, further widening the gap between human AD pathology and its most commonly used models.

In fact, there is growing evidence of very early involvement of other mechanisms which may commence even before the emergence of tau and Aβ pathologies in LOAD pathogenesis [5,8]. These potentially triggering mechanisms include but are not limited to vascular pathology [9], mitochondrial dysfunction [10], oxidative stress [1], hypoxia [11], insulin resistance [12], and chronic neuroinflammation [13].

It has been proposed that a combination of chronic neuroinflammation and (pathological) aging, the so-called ‘neuroinflammaging’ state [14,15], plays a major role in the mechanism of neurodegenerative disorders, including AD [16,17]. Notably, genetic variants associated with the regulation of innate immunity and phagocytosis (for example, TREM2 [18,19] or CD33 [20]) have been identified as risk factors for LOAD [21,20]. Similarly, neuropathological studies have supported early involvement of neuroinflammation in AD through demonstrating the accumulation of activated microglia and inflammatory mediators in the cerebral neocortex at a low Braak stage for AD pathology [22].

In this study, we focused on characterizing the models suitable for studying the inflammation hypothesis of Alzheimer’s disease, based on which neuroinflammation is considered as the driving force of AD pathology and starts early in the course of the disease, prior to tau hyperphosphorylation and amyloid plaque formation [23,13]. A thorough characterization of these models will enable future research to understand the possible transition from ‘neuroinflammaging’ state to neurodegeneration and also to test the efficacy of potential therapeutic agents to prevent such a transition.

### Neuroinflammation in Alzheimer’s disease

Neuroinflammation is known as a key component in the neurodegenerative process of Alzheimer’s disease [24]. Characteristics of neuroinflammation, like severity and duration, vary depending on the underlying causes. At one end of the spectrum, there are autoimmune disorders, such as multiple sclerosis, featuring chronic neuroinflammation mainly driven by Th1 cells (reviewed in [25]); At the other end of the spectrum, there is a less fulminant ‘smoldering’ form of chronic neuroinflammation driven by cells of the innate immunity ([26], reviewed in [27]). The latter is mainly due to age-related impairment of anti-inflammatory mechanisms that leads to the aforementioned ‘inflammaging’ state [18,20,28] and causes subtle clinical symptoms, as exemplified by neuroinflammation following traumatic brain injury, which may persists for years prior to clinical manifestation as AD [29].

The most recent perspective of the inflammation hypothesis of LOAD, proposed by Krstic et al. [13], has provided a comprehensive sequence of pathological events leading to AD pathology. Based on this hypothesis, the natural neuronal response to inflammatory stress includes hyperphosphorylation of tau (hp-Tau) and mislocalization of hp-Tau towards the somatodendritic compartment as well as increased APP synthesis [13]. Under physiological conditions, the resulting APP aggregates are cleared by neuroprotective microglia [13]. However, in the setting of pathological aging, for example, midlife overweight and obesity [30], microglia become hyper-reactive with increased release of pro-inflammatory cytokines and dysfunctional phagocytosis [13]. This results in further exposure of neurons to a neurotoxic pro-inflammatory environment without the guard of neuroprotective microglia [13]. The consequent neuronal injury includes breakdown of the axonal cytoskeleton leading to the impairment of axonal transport, formation of axonal swellings of APP aggregates [13], and eventually dystrophic neurites that cannot be removed by hyper-reactive microglia [13]. Secondary to this neuronal degeneration, Aβ plaques are formed from the intracellular APP aggregates [13]. These plaques will trigger further release of pro-inflammatory molecules leading to a vicious circle of neurotoxic pro-inflammatory response [13].

The pathological activation of microglia, which is the center of this proinflammatory response, is characterized by upregulation of MHC antigens and complement receptors [31], as well as release of various pro-inflammatory factors like tumor necrosis factor-α (TNF-α), interleukin-1 β (IL-1β), IL-6, and reactive nitrogen and oxygen species [31]. These pro-inflammatory factors are neurotoxic especially if accumulated during a chronic neuroinflammatory process [31].

Alternatively, some microglia deteriorate in the process of immune system senescence [32]. Histopathologically, this status manifests with microglial dystrophy, which is distinguished from cytoplasmic hypertrophy as seen in activated microglia. Dystrophic microglia are also associated with neurofibrillary degeneration in AD brain, especially in the temporal lobe [33]. Such observations have led to the hypothesis that senescence of microglia itself might be the initial trigger of Alzheimer’s disease neuropathology; in this regard, Alzheimer’s disease would be viewed as an immune senescent rather than neuroinflammatory condition [33,32]. However, growing evidence suggests that hyper-reactive microglia is involved in early stages of LOAD [34], but may more rapidly undergo the process of senescence, and thus become non-functional after the initial induction of an aberrant inflammatory response. It is noteworthy that in such dystrophic status, like hyper-reactive mode, microglia remain unable to fulfill their physiological roles of clearing the neurotoxic aggregates [35], like Aβ oligomers, and producing neurotrophic factors, therefore, allowing the process of neurodegeneration to progress. Such paradigm may explain the early-stage responsiveness of the disease to NSAIDS compared to its NSAIDS-induced aggravation at later stages. Restraining microglial activity in early stage will slow the disease; but in later stages, it will accelerate the disease process probably through restraining the residual neuroprotective and clearance function of dystrophic microglia [36].

### Rodent models of neuroinflammation

In conventional transgenic animal models of AD, neuroinflammation is mainly known as a secondary response to sustained Aβ overproduction and deposition. It includes microglial activation and variable involvement of the complement system and production of cytokines [13,17,37]. Altogether, in these models, the inflammatory response is incomplete and less severe compared to AD in humans [13]. Janelsins and colleagues detected early activation of inflammatory processes in the entorhinal cortex (but not hippocampus) of the triple transgenic model (3xTg) of AD at 3 months of age [38]. Interestingly, the neuroinflammation process was concurrent with the production and accumulation of intracellular Aβ but occurred prior to any significant extracellular Aβ plaque deposition, which manifests at about 12 months of age in the 3xTg mice [38]. Of note, this neuroinflammatory process was characterized by a selective trend of increasing expression of TNF-α and monocyte chemoattractant protein-1 (MCP-1), which was not detected for 21 other cytokines tested [38]. Moreover, a substantial microgliosis was detectable at 6 months of age. Although this study provided valuable evidence for a contributory role of inflammatory factors like TNF-α and MCP-1 in AD pathology, the model system replicates the familial but not sporadic type of AD [38].

An ideal disease model should recapitulate causes, lesions, and symptoms in a chronological order similar to the actual disease [7]. A faithful model to the inflammation hypothesis of AD should be an aged animal that recapitulates early chronic neuroinflammation prior to hyperphosphorylation of tau and Aβ plaque deposition. In rats, a neuroinflammatory process lasting more than 7 days is considered chronic neuroinflammation [39]; and rodents older than 22 months are considered senescent [40].

**Figure 1:**
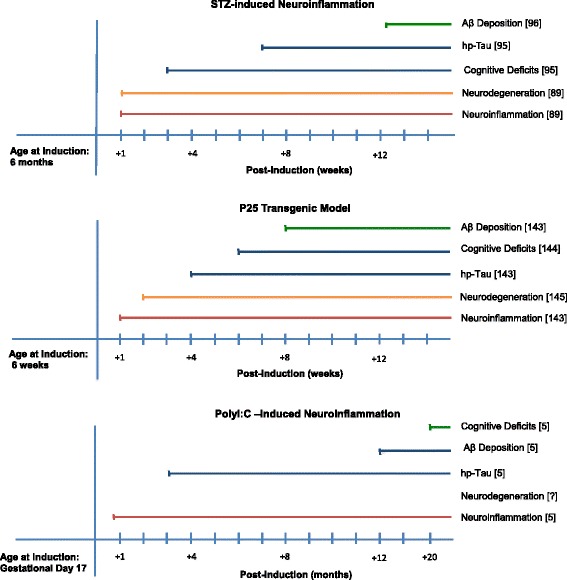
Time course of pathological events in models compatible with inflammation hypothesis of Alzheimer’s disease. In the models shown, neuroinflammation starts prior to the appearance of AD related lesions (hp-Tau and Aβ depositions). Animals develop cognitive deficits at variable time points after the induction of neuroinflammation in the respective models. In contrast to the most of transgenic AD animal models, the STZ and p25 Tg models of neuroinflammation feature neurodegeneration. It is noteworthy that the PolyI:C model has the longest time lapse between induction of neuroinflammation and cognitive deficits. Note that the time points do not necessarily represent the actual time of appearance, but the time points when the pathological hallmarks were detected in the respective references (Abbreviations: *LPS* lipopolysaccharide; *PolyI:C* polyriboinosinic-polyribocytidilic acid; *p25 Tg* p25 transgenic model; *IL-1β Tg*: interleukin-1β transgenic model; *ICV-STZ* intracerebroventricular streptozotocin model; *hp-Tau* hyperphosphorylated tau; *Aβ* amyloid-β).

Here, we reviewed potential rodent models of AD that present early neuroinflammation in the disease process and are not genetically manipulated by mutations related to Aβ or tau production (summarized in Table 1). In this regard, inflammatory responses in amyloid-injected models (reviewed in [41]) are beyond the theme of this article. We categorized models, based on the mechanism of their creation, to immune challenge-based, toxin-induced, and (non-AD) transgenic models. Current knowledge on the chronology of pathological events was analyzed for each model to discuss its potential compatibility with the inflammation hypothesis of AD (see Figure 1 for the compatible models).

**Table 1:** **Rodent models of neuroinflammation**

**Models**	**Predisposing factors/causes**	**Time of appearance of lesions**		**Signs (time detectable)**	**SLC reading key**	**Reference**
		**hp-Tau**	**Aβ depositions**			
LPS	Peripheral immune challenge, chronic neuroinflammation	?	?	Fear memory (?)	S1L0C1	[165]
				Spatial memory (?)		
PolyI:C	Peripheral immune challenge, chronic neuroinflammation	3m	12m	Spatial memory (20 m)	S1L1C1	[5]
		(PHF, but not NFTs)	(APP depositions)			
ICV-STZ	Disrupted insulin signaling, chronic neuroinflammation	6-7w	12w	Spatial memory	S1L1C1	[85]
				Visual recognition memory (3w)		
ICV-OKA	Inhibition of serine/threonine phosphatases 1 and 2A	2w	6w	Spatial memory (?)	S1L1C0	[102] [104]
		(PHF, but not NFTs)	(Non-fibrillar Aβ deposits)			
ICV-colchicine	Inhibition of tubulin formation/microtubule breakdown	? (Tau dephosphorylation)	? (Amyloid plaque)	Spatial memory (14d to 21d)	S1L0C1	[113] [117]
p25 Tg	Upregulation of cPLA2, neuroinflammation	4w	8w	Contextual fear memory (6w)	S1L1C1	[145]
IL-1 β Tg	Chronic neuroinflammation	?	? (Increased clearance of amyloid plaques)	Contextual fear memory (12w)	S1L0C0	[39]
Anti-NGF antibody Tg	Blockade of NGF signaling pathway	? (Neurofibrillary pathology)	? (Amyloid plaques)	Visual recognition memory (4 m); Spatial memory (9 m)	S1L1C0	[148] [149]

This table summarizes the suggested models of late-onset AD (LOAD) displaying neuroinflammation as one of the prominent pathological events. The SLC reading key is a scoring system that represents the compatibility of an animal model with the disease in humans with respect to signs (S), lesions (L), and causes (C) [7]. Compatibility is indicated by 1 and incompatibility by 0. Based on SLC reading key, p25 tg, PolyI:C-, and STZ-induced neuroinflammation models are compatible with the inflammation hypothesis of LOAD [13]. (Abbreviations: ? unavailable data; *LPS* lipopolysaccharide; *PolyI:C* polyriboinosinic-polyribocytidilic acid; *p25 Tg* p25 transgenic model; *NGF* nerve growth factor; *IL-1β Tg* interleukin-1β transgenic model; *ICV* intracerebroventricular; *STZ* streptozotocin; *OKA* okadaic acid; *hp-Tau* hyperphosphorylated tau; *Aβ* amyloid β; *PHF* paired helical filaments; *NFT* neurofibrillary tangles; *cPLA2* cytosolic phospholipase 2; *SLC* Signs, Lesions, Causes; *w* week; *m* month).

### Immune challenge-based models

#### Lipopolysaccharide (LPS)-induced neuroinflammation

A commonly studied model of neuroinflammation is LPS-induced neuroinflammation which represents the current standard paradigm to study neuroinflammation both *in vivo* [42,43] and *in vitro* [26,44]. LPS, also known as endotoxin, is a component of the outer membrane of gram-negative bacteria. LPS binds CD14 on microglia membranes. The LPS-CD14 complex then interacts with the toll-like receptor-4 (TLR-4) [26,45], which, in turn, activates microglia by initiating signal transduction cascades leading to rapid transcription and release of pro-inflammatory cytokines [46] (including IL-1, IL-6, IL-12, p40, and TNF-α), chemokines (for example, CCL2, CCL5, and CXCL8), the complement system proteins (for example, C3, C3a, and C5a receptors) [46], as well as anti-inflammatory cytokines like IL-10 [47] and transforming growth factor-β (TGF-β) [48].

Different paradigms of LPS-induced neuroinflammation exist with respect to the route of administration, duration of exposure and age of the animals [49]. While chronic central administration of LPS can induce memory and learning deficits analogous to AD cognitive decline [50], systemic LPS administration led to selective hippocampal impairment in context-object discrimination but not spatial memory [51]. Moreover, Bordou and colleagues recently investigated the role of duration of exposure to LPS as well as the age of exposed rats on the neuroinflammatory response to LPS. Male rats at three age groups of young (3 months), middle-aged (9 months), and aged (23 months) received continuous infusion of picomolar levels of LPS (or artificial CSF as control) into their fourth ventricle [49]. The duration of exposure was either 3 or 8 weeks. Among all cytokines, TNF-α increase in response to LPS infusion was similar in different age groups [49]. However, in contrast to young rats, IL-1β did not significantly increase after 3 weeks of infusion in middle-aged and aged rats. Instead, aged rats had significantly increased IFN-γ compared with younger rats [49]. Among rats of the same age group, longer duration of exposure to LPS infusion significantly increased the elevations of IL-1-α, IL-2, IL-4, IL-5, IL-6, IL-12, IL-13, and GM-CSF levels [49]. This study provides an evidence of the influence of age and chronicity of infection on neuroinflammatory responses in certain regions of the brain, like locus coeruleus, which undergo significant cell loss in early stages of AD [52].

In similar studies performed by Wenk and coworkers [43,53-55], chronic neuroinflammation was modeled through continuous infusion of picomolar concentrations of LPS into the fourth ventricle of adult rats. A widespread activation of microglia was detected 2 days after the initiation of LPS infusion [55]. Within 2 weeks after the cessation of LPS infusion, microglial activation decreased in most brain areas barring the hippocampus, and after the following 2 weeks, inflammation was mainly localized in the hippocampus [55]. Furthermore, MRI scans showed shrinkage of the temporal lobe and enlargement of the lateral ventricles. Of note, electron microscopic studies showed impaired protein synthesis in hippocampal neurons of LPS-injected animals [54]. Moreover, neuronal loss and impairment of long-term potentiation were reported in the entorhinal cortex [56] and the dentate gyrus of the hippocampus respectively [57], altogether explaining the decline in spatial memory [56]. In this model, LPS-induced neuroinflammation was time dependent (maximal within 4 weeks of infusion) as well as cell and region specific (microglia in hippocampus) [55].

Other groups have provided evidence of exacerbated AD-related protein pathology such as increased Aβ production through enhanced β-secretase activity in APP sweTg [44] and tau hyperphosphorylation in 3xTg-AD mice following LPS injection [42]. However, wild-type animals injected with LPS showed no increased Aβ deposition in the time course of 3 months. The authors argue that this process might occur at a later time point due to severe neuronal dysfunction and neurodegeneration [44]. Another explanation for the lack of Aβ deposition in LPS-injected animals was proposed by DiCarlo et al. by showing a reduction of established Aβ plaques after intrahippocampal LPS injection through stimulation of Aβ clearance [58].

In line with neurodegenerative structural changes, LPS-induced neuroinflammation caused cognitive deficits leading to impaired performance in associative and spatial learning tasks [59,60]. Finally, a long-term characterization of LPS-induced changes with regard to the chronology of histological and ultrastructural changes, as well as cognitive deficits, is required to assess the compatibility of LPS neuroinflammation model with the inflammation hypothesis of AD [13].

### PolyI:C-induced neuroinflammation

It is well understood that peripheral infections influence the inflammatory state of the central nervous system [61,62]. The brain innate immune system reacts to systemic inflammation through activation of microglia (reviewed in [13,31]), which may become a potential trigger for neurodegenerative processes [31,61], especially when neuroinflammation becomes chronic in an aging brain [31]. Based on this evidence, a recent study proposed a non-transgenic mouse model of LOAD featuring chronic neuroinflammation after *in utero* systemic immune challenge [5]. Polyriboinosinic-polyribocytidilic acid (PolyI:C) is a synthetic double-stranded RNA that induces an innate immune response analogous to acute viral infections in treated animals. In the CNS, this immune response is mainly mediated by TLR-3-induced activation of microglia [63], followed by an NF-kB-dependent induction of proinflammatory cytokines including IL-1β, IL-6, IL-8, TNF-α, and type I and II interferons [64].

Exposure to PolyI:C leads to inflammation in the injected animal, as well as a chronic proinflammatory state in the fetus of pregnant animals [5,65,66]. Systemic injection of PolyI:C on gestational day 17 led to chronic neuroinflammation, as observed by significantly higher levels of IL-1 and IL-6 compared to the control group [5]. Increased brain cytokine levels were detectable as early as the age of 3 weeks and sustained throughout aging [5]. However, tau hyperphosphorylation started only after 3 months of age and, in spite of some fluctuation, was significantly higher than controls at 6 and 15 months of age [5]. Amyloidogenesis started as late as 12 months of age [5]. Later, at 20 months, animals in the immune-challenged group presented with a significant impairment of spatial recognition memory compared to the control group.

There is growing evidence that early-life infection may lead to abnormalities in cognitive aging [67], probably due to glial priming effect, leading to significantly enhanced glial reactivity to a subsequent immune challenge later in life [67]. The late-gestational PolyI:C induced neuroinflammation model is an informative approach to study the effects of life-long neuroinflammation on cognitive function. Notably, a single intraperitoneal injection of the pregnant mouse was sufficient to change the inflammatory state of the progeny throughout the entire life span, with two additional PolyI:C injections in adulthood exacerbating the pathology. Further work is required to more thoroughly assess the cognitive phenotype of the aged animals, especially since Bitanihirwe et al. described neuropsychiatric changes in prenatally stimulated adults [68].

It is noteworthy that the APP deposition and paired helical filaments (PHF) in this model do not completely replicate the lesion in advanced human AD pathology, that is, Aβ plaques and neurofibrillary tangles (NFT). However, the protein pathology is entirely endogenous and not relying on overexpression of human proteins in murine cells. Thus, the differences between PolyI:C-challenged mice and human AD patients might reflect characteristics of the respective species. Finally, the chronological order of pathological events and cognitive deficits in the PolyI:C model is compatible with the inflammation hypothesis of AD [13]; and therefore, it may be a suitable model for studying early stages of LOAD pathology from this perspective.

### Neurotoxin-induced models

#### Streptozotocin-induced neuroinflammation

Since Siegfried Hoyer [69,70] and Suzanne Craft [71] first described the causality between impaired insulin signaling and cognitive dysfunction, a growing body of evidence has emerged on impaired brain insulin signaling and glucose metabolism in LOAD (reviewed in [72]). Peripheral injection of the glucosamine-nitrosurea compound streptozotocin (STZ) selectively damages pancreatic β-cells after being taken up via the glucose transporter SLC2A2 [73,74]. Thus, repetitive intraperitoneal injection of STZ is an established animal model of diabetes mellitus [75]. Interestingly, after STZ-mediated induction of diabetes, rodents display impaired neuronal plasticity and learning deficits [76]. In a recent study, STZ-induced diabetic rats presented frontal lobe neurodegeneration (as evidenced by FJC staining) and hippocampal atrophy, accompanied by Aβ aggregation, synapse loss, and the consequent cognitive decline 4 months after receiving IV STZ [77]. These deficits are mainly induced by peripheral effects of STZ as the molecule does not cross the blood-brain barrier [78,79].

Acutely, STZ causes oxidative stress through the generation of hydrogen peroxide [80] and NO [81]. Furthermore, it leads to DNA damage by alkylation and methylation leading to apoptosis [82]. Notably, since PARP knockout animals are resistant to STZ-induced diabetes, cell death is likely caused by depletion of reduction equivalents followed by ATP depletion [83]. In the long term, STZ induces a state of metabolic imbalance characterized by impaired insulin secretion (reviewed in [78]) and inflammation [84]. Intracerebroventricular (ICV) [85-87] or intracerebral [88] STZ injection induces impaired brain insulin signaling in rodents. Regardless of the route of administration, the animals develop neuroinflammation and cognitive deficits. The following discussion focuses on the ICV injection of STZ.

A single ICV injection of 1 or 3 mg/ml STZ in rats has been shown to cause chronic neuroinflammation, dilation of the ventricles, and atrophy of the septum with reduction of neuronal cell counts [89]. Both STZ concentrations cause these effects; however, they are more pronounced at 3 mg/ml [89].

When administered to transgenic models of AD, ICV-STZ was shown to exacerbate neuroinflammation, cognitive deficits, plaque pathology, and tau hyperphosphorylation [90,91], indicating that STZ renders the brain more susceptible to the pathological hallmarks of AD. Similar exacerbating effects were observed when STZ was administered intraperitoneally to AD transgenic mice [92-94].

Chen and coworkers have recently compared ICV-STZ wild-type and the widely used 3xTg AD mouse models [85]. Immunohistochemical studies showed early and extensive neuroinflammation in the ICV-STZ mice, characterized by increased astroglial and microglial activation especially in hippocampal CA1, CA3, and dentate gyrus [85]. STZ-induced neuroinflammation was more pronounced as compared to neuroinflammation in 3xTg mice in the same regions [85].

In the ICV-STZ wild-type model, neuroinflammation was detectable 1 week after low-dose ICV-STZ injection while paraventricular Aβ depositions and hippocampal hyperphosphorylated tau appeared within 3 months of ICV-STZ injection [89,85]. With high-dose ICV-STZ injection, however, tau hyperphosphorylation was detectable as early as 4 weeks in rats [95] and 6 weeks in mice [85]. Within 3 weeks of STZ injection in wild-type mice, spatial and short-term memory deficits developed, indistinguishable from cognitive deficits in 3xTg mice.

All in all, the ICV-STZ model not only displays neuroinflammation but also reproduces tau [85] and amyloid [89] pathologies as well as AD-like cognitive deficits [96,97] with a chronology compatible with the inflammation hypothesis of AD [13].

#### Okadaic acid-induced neuroinflammation

A comparable model to STZ-induced neuroinflammation is okadaic acid (OKA)-induced neuroinflammation. OKA is a major polyether toxin that selectively inhibits serine/threonine phosphatases 1 and 2A [98]. The decreased activity of protein phosphatase 2A (PP2A) has been observed in the pathology of AD [99] and was proposed to be involved in hyperphosphorylation of tau [100].

In line with the abovementioned molecular link to AD pathology [101], (ICV) OKA injection develops memory impairment in rats [102,103], making it suitable for further characterization as a potential AD model [104]. In studies performed by Arendt and colleagues [105], ICV infusion of OKA (70 ng/day; for up to 4 months), could replicate some AD-associated pathologies including hyperphosphorylation of tau (at Ser-202/Thr-205) and apoptotic cell death within 2 weeks, as well as cortical deposition of non-fibrillar Aβ within 6 weeks of infusion. Interestingly, Lee and colleagues later confirmed the formation of paired helical filaments of tau following intra-hippocampal injection of OKA (1 mM, 0.5 ml) [106]. It is noteworthy, however, that in this model, hyperphosphorylated tau aggregates do not develop into NFTs [105].

In addition to AD-like histopathological changes, in a recent study, memory impairment was reported in the Morris water maze test 15 days after ICV-OKA (200 ng) injection [107]. In contrast to control and vehicle groups, OKA 200 ng treated rats did not present significant decrease in latency time to reach the platform in the second and third sessions as compared to the first session [107].

Interestingly, OKA-induced memory impairment is found to be associated with neuroinflammation [108]. In OKA-injected rats, neuroinflammation was characterized by increased expression of proinflammatory cytokine TNF-α and IL-1β as well as total nitrite in both hippocampus and cortex [107].

However, the effect of antidementic (non-antiinflammatory) drugs on subsiding the neuroinflammation supports the reactive rather than the causative role of neuroinflammation (with regard to neurodegeneration) in this model [109,103]. Thirteen days of pretreatment of OKA-injected rat with anti-dementic drugs memantine (10 mg/kg) and donepezil (5.0 mg/kg) could protect not only the ICV-OKA-induced memory impairment but also the associated changes in TNF-α, IL-β, and total nitrite levels as well as expressions of iNOS and nNOS [107,110]. In addition, recent studies have shown that OKA-induced oxidative stress [111,112] is linked to dysfunction of astrocytic neuroprotection [102]. Twelve days after intra-hippocampal injection of OKA (100 ng), rats developed spatial cognitive impairment, accompanied by hippocampal astrogliosis (as evident by, increased GFAP), and oxidative stress (for example, decreased glutamine synthetase and decrease in reduced glutathione content) [102]. Thus, the application of this model as an etiology-based model of neuroinflammation in LOAD requires further characterization of the sequence of different pathological events, including the possible precedence of oxidative stress.

#### Colchicine-induced neuroinflammation

Similarly, ICV injection of colchicine in rats could induce AD-like pathology with consequential cognitive and behavioral alterations similar to AD [113]. Colchicine is a cytotoxic agent that irreversibly binds to tubulin molecules and thus halts the aggregation of tubulin dimers to the fast growing end, causing disruption of microtubule polymerization [114]. Blocking axoplasmic transport, colchicine severely damages hippocampal granule cells and mossy fibers, eventually leading to neuronal loss, which manifests with cognitive impairment and spontaneous motor activity [115]. Systemic and neurologic symptomatology of rats in response to the central administration of high-dose colchicine is further detailed in [113].

Neuroinflammation plays a key role in the development of AD-like neurodegeneration in this model [113,116,117]. In a recent study, Ho and colleagues found both *in vitro* and *in vivo* evidence for the involvement of COX-2-mediated apoptotic mechanisms in colchicine-induced neurotoxicity [116]. After intra-hippocampal injection of colchicine in rats, a significant increase in COX-2 mRNA levels was found in dentate gyrus granule cells, followed by apoptotic morphological changes [116]. Similarly, Sil et al. demonstrated that colchicine-induced neurodegeneration is mediated by COX-induced neuroinflammation [117]. In this study, the TNF-α level of the hippocampus was significantly higher in the colchicine-injected (15 μg, ICV) rats compared to control and sham-operated rats. Likewise, the nitrite level and the ROS level of hippocampus were also significantly higher in colchicine-induced neurodegeneration compared to controls and sham-operated rats [117]. This study also reported significant increase in amyloid plaques found in the hippocampus. It is noteworthy that naproxen administration (doses 5, 10, or 20 mg/kg) could prevent from TNF-α increase and reduce the amyloid plaque formation [117].

Of note, AD-like tau pathology, however, does not occur in colchicine model [118]. In fact, although colchicine leads to microtubule breakdown [118], as seen in Alzheimer’s disease, its mechanism is based on tau dephosphorylation rather than hyperphosphorylation [119,120]. The chronological association of the pathological events with neuroinflammation is yet to be investigated in this model. Without chronological characterization, it is unclear if this model is compatible with the inflammation hypothesis of AD.

### Genetically manipulated models (unrelated to mutations in familial AD)

#### IL-1β overexpression model

Interleukin-1β (IL-1β), regulating acute and chronic neuroinflammatory responses (reviewed in [121,122]), is found elevated in AD patients [123,121,124,125]. Based on this notion, O’Banion and coworkers have developed an inducible IL-1β overexpression model of chronic neuroinflammation (IL-1β excisional activation transgenic (XAT) mouse model) [126]. Prolonged IL-1β elevation induces microgliosis and astrogliosis alongside with chronic elevation of the proinflammatory cytokines, IL-6 and TNF-α [126]. After activation of the inducible transgene in this model, neuroinflammation may last as long as 10 months [126].

IL-1β transgenic mice displayed a dualistic histopathological presentation with respect to the hallmarks of AD. On the one hand, APP production and processing are unaltered despite prolonged overexpression of the transgene [126]; and notably, the amyloid plaque burden was even reduced in crossed IL-1β XAT and APP/PS1 Tg mice [126,127]. On the other hand, crossing IL-1β XAT and 3xTg AD mice led to significant exacerbation of tau hyperphosphorylation within 1 month of IL-1β overexpression [128]. It is conceivable that in this model, IL-1β per se induces activation of microglia without shifting it to a dysfunctional hyper-reactive state, therefore sparing its ability to readopt the phagocytic mode in the aid of clearance of Aβ plaques [129].

It is likewise noteworthy that the IL-1β XAT mouse model lacks overt neuronal loss or apoptosis within 2 and 5 months of IL-1β overexpression, respectively [127,126]. However, despite the absence of neuronal loss, the animals manifested significant cognitive deficits, including contextual fear memory and spatial memory defects within 3 months of transgene induction [39].

These divergent effects of IL-1β overexpression on Aβ and tau pathology can be explained in the context of inflammation hypothesis of AD proposed by Krstic and Knuesel [13]. Based on this hypothesis, tau hyperphosphorylation is an early neuronal response to neuroinflammatory stress, while Aβ pathology emerges after microglial shift to the pro-inflammatory M1 phenotype as opposed to the phagocytic M2 phenotype [13]. This implies that in the IL-1β XAT mouse model, microglia maintain their physiological function [130] in contrast with immune challenge-based animal models (for example, LPS and PolyI:C injection) or neurotoxin (that is, STZ) models.

Altogether, in spite of corroborating the association between chronic neuroinflammation and cognitive deficits, the IL-1β model could not reproduce the main lesions of AD pathology; therefore it may not be a suitable model for sporadic AD.

#### p25 transgenic model

Neurons are considered as terminally differentiated non-dividing cells. However, the evidence of expression of cell cycle-specific proteins [131] and DNA replication in neurons prior to neurodegeneration in AD-prone brain regions [132-134] supports the association of AD neurodegeneration with cell cycle dysregulations. Regulation of the cell cycle is performed by cyclins and cyclin-dependent kinases (CDKs) (reviewed in [135]). CDK5, in particular, also plays an important role in the brain development by promoting neurite outgrowth in post-mitotic neurons [136,137]. p35 is a regulatory subunit of CDK5 [138], and cleavage of p25 by the calcium-dependent kinase calpain leads to neurotoxicity through aberrant CDK5 activation [139]. Interestingly, neurotoxicity itself drives p35 cleavage possibly creating a vicious cycle [140].

CDK5 activation and aberrant p25 accumulation was shown in AD patients [139]. Notably, mice overexpressing human p25 displayed AD-like pathology [141], suggesting p25 overexpression as a potential mechanism to model AD [142]. Unlike its precursor p35, the p25-CDK5 complex was shown to induce tau hyperphosphorylation possibly explaining the corollary AD-like pathology [141]. In p25 Tg mice, neuroinflammation is detectable before other AD pathology hallmarks, including tau hyperphosphorylation [143]. Said neuroinflammation is characterized by astrocytosis and increased levels of pro-inflammatory cytokines, like TNF-α, IL-1β, and MIP-1α [143]. Activation of microglia was also detectable 4 weeks after induction of p25 overexpression [143].

Interestingly, Sundaram and colleagues reported a relatively clear temporal sequence between neuroinflammation and secondary pathological hallmarks associated with AD [143]. While neuroinflammation occurred as early as first week, tau hyperphosphorylation and amyloidogenesis were detectable at 4 and 8 weeks after p25 induction, respectively [143]. Finally, cognitive deficit (contextual fear memory) was detectable within 6 weeks of p25 induction [144].

In summary, the p25 Tg mice resemble the histopathological hallmarks of AD with amyloid depositions [143], tau hyperphosphorylation [143], and neurodegeneration [145]. Additionally, these mice display cognitive deficits [144]. These pathological events occur in a chronological order compatible with the inflammation hypothesis of AD [13].

#### Anti-nerve growth factor (NGF) transgenic models

The transgenic expression of anti-nerve growth factor (NGF) antibodies led to an overt neurodegenerative phenotype in aged mice [146,147]. This model, known as AD11 model, resembles the insidious cognitive decline of LOAD by manifesting with a significantly progressive deficit in visual recognition memory (as evaluated through an object recognition test) and spatial memory (as evaluated through an eight-arm radial maze), starting at 4 months and 9 months of age, respectively [148]. Of note, in this model, neurodegeneration was characterized by neuronal loss, cholinergic deficit, tau hyperphosphorylation (associated with neurofibrillary pathology) and Aβ plaques [149].

The primary assumptions alluded to a link between chronic deprivation of NGF and abnormal processing of amyloid precursor protein, leading to Aβ excessive formation and deposition [149,150]. However, growing evidence, including gene expression profiles [151], is showing early involvement of neuroinflammatory elements in AD11 model [152]. Interestingly, changes in the expression of inflammatory and immune response genes were the earliest and most significant [151]. Specifically, significant changes were found in the expression of genes encoding for proteins of the complement cascade and the major histocompatibility complex (MHC). D’Onofrio and colleagues demonstrated that in AD11 model, overexpression of C3 mRNA was significantly high as early as P30 in the cortex and hippocampus and at P90 in all brain areas [151]. Also, C1qb was significantly upregulated in the cortex and hippocampus at P90. Similarly, a significant dysregulation of MHC class I gene expression was observed in AD11, as some mRNAs of Class I MHC (in particular H2-Q1) were severely reduced in some areas at P30 but significantly increased at P90 [151]. This dysregulated MHC class I expression may explain the disruption in dynamic synaptic strength and connectivity, processes involved in memory formation.

Beside the above-explained ‘non-immune’ involvement of inflammatory factors in AD11 models, there is growing evidence on their role in a concurrent neuroinflammation process, further aggravating neurodegeneration. For example, the gene expression profiles showed significant changes in the expression levels of other inflammatory-related mRNAs, such as Ccl5 (chemokine ligand 5)/RANTE, IL-1β, TNF-α, IFN-γ-induced ATPase, Cd47, Ccl17, Cd300lf, Cd72, and Cox-2 [151]. Altogether, AD11 mice demonstrate a complex neurotrophic and inflammatory dysregulation in key brain regions with potential role in the outset of the neurodegeneration process described in this model [146]. However, in order to avoid probable autoimmune reactions and achieve a more brain-specific phenotype, an inducible NGF knockout might be a more relevant approach.

Similarly, inflammatory response gene expression was significantly activated in AD10 mice [153], a variant of AD11 model without the antibody heavy chain, leading to a similar neurodegenerative picture [154]. Interestingly, however, when AD10 mice were housed in a murine pathogen-free environment, the inflammatory gene response significantly subsided, and neither an overt neurodegeneration nor behavioral symptoms occurred [154].

Histopathological studies that characterize the microglial activation in this model are lacking. Of special interest would be the temporal relationship of neuroinflammation and other AD neurodegeneration processes, including tau pathology and amyloid deposition. Thus, the compatibility of this model with the neuroinflammation hypothesis of AD is yet to be investigated.

#### TGF-β-deficient models

TGF-β is a cytokine involved in several opposing physiological functions in inflammation pathways and cell growth, depending on the target cell type, cell environment, as well as amount and duration of exposure to TGF-β [155,24,156]. In the CNS, TGF-β is produced by both neurons and glial cells [157]. Recent evidence from autopsied samples of AD patients showed elevated TGF-β in brain microvessels, leading to the release of pro-inflammatory cytokines like TNF-β and IL-1β from endothelial cells in the brain [158]. This is in line with experimental findings on TGF-β transgenic mice, showing that long-term increase in expression of TGF-β is associated with increased perivascular amyloidogenesis [156]. On the other hand, short-term increase in TGF-β expression was neuroprotective [156]. Notably, a modest increase of astroglial TGF-β1 expression enhanced Aβ clearance in aged human APP transgenic mice [159], confirming the neuroprotective role of TGF-β1. Thus TGF-β knockout models, completely lacking the mentioned neuroprotective effects, have demonstrated overt neurodegeneration (reviewed in [24]). TGF-β-/- mice displayed neurodegeneration and neuroinflammation at P1 and P21 without a clear temporal sequence of the two events [160]. However, in order to create a model based on TGF-β1 deficiency to study the inflammation hypothesis of AD, the effects of TGF-β1 deficiency (and thus its neuroprotective function) on neuroinflammatory dysregulations and its potential effect on triggering AD-like neurodegeneration in aged rodents should be investigated.

## Discussion

The focus of this study was to recognize rodent models of LOAD that present a process of chronic neuroinflammation prior to tau and amyloid pathology, as described by the inflammation hypothesis of AD [13]. Among models of chronic neuroinflammation, here we found ICV-STZ and PolyI:C-induced neuroinflammation and p25 transgenic (p25 tg) models most compatible with the inflammation hypothesis of AD in terms of temporal ordering of pathological events. The evidence on temporal order of pathological events is more controversial in LPS- and OKA-induced neuroinflammation. The timeline of AD-related pathological events in colchicine-induced neuroinflammation as well as anti-NGF overexpression and TGF-β1 knockout models is yet to be investigated. Owing to the clear temporal order of pathological events, ICV-STZ, PolyI:C, and p25 tg models are more suited to study the effect of anti-inflammatory agents on different stages of LOAD; the results of such studies may improve the design of clinical trials for potential preventive or therapeutic agents.

Since most cells (for example, microglia) and molecules (for example, TNF-α, TGF-β) of the immune system are multifunctional [161] and sometimes even demonstrate completely opposite functions, depending on the context [161], neuropathological characterization of neuroinflammation provides little information on its actual role in AD pathogenesis [24]. In addition, while temporal precedence of neuroinflammation supports its causative role in neurodegeneration [17], it does not rule out a physiological neuroprotective role of an early neuroinflammatory process against an underlying pathological process [161]. Of course, a physiological role is much less probable in the case of chronic neuroinflammation which by definition implies the inability of the immune system to completely remove or deactivate the injurious agent and then resolve [17].

The detrimental role of chronic neuroinflammation is more clear in those models based on direct stimulation of the immune system (that are, LPS, PolyI:C, and Il-1beta overexpression model) [161]. For models not based on direct stimulation of immune system, further evidence is required to analyze the actual role of neuroinflammation in respect to each stage of AD pathology. For instance, studies that demonstrated reduced AD-related symptoms and pathology in animals pretreated with anti-inflammatory drugs support the pathological role of neuroinflammation in those models. For example, Dhull and colleagues reported significant increase in survival of hippocampal neurons and improvement in memory performance (as tested in Morris water maze) in ICV STZ rats who received COX-1 and Cox-2 inhibitors [162]. Similarly, administration of naproxen (a nonspecific COX inhibitor) reduced the amyloid plaque formation in the ICV colchicine-induced neuroinflammation model in a dose-dependent manner [117].

Neuropathologically, AD is characterized by extracellular amyloid plaques and intracellular neurofibrillary tangles [17,163]. Although amyloid or tau pathology followed chronic neuroinflammation in the above-discussed models, formation of typical senile plaques and neurofibrillary tangles, as seen in human AD, was not observed in these studies. Instead, intermediate pathological species, like Aβ aggregates [143] and hyper-phosphorylated tau [97,141], were considered as signs of amyloid and tau pathologies, respectively. Of note, the recapitulation of these pathological hallmarks is a great challenge in every rodent model of AD [164].

Moreover, among the discussed studies, different types of molecular evidence were considered as the sign of AD-like amyloid pathology, including Aβ plaque-like structures in STZ-induced model [96], APP containing plaques in PolyI:C-induced model [5], increased levels of alpha-, beta-, and gamma-secretase activity in brain lysate, and intracellular Aβ aggregates in LPS model [50]. Similarly, the evidence of neurodegeneration was also different among these models; for instance, LPS-induced model led to apoptotic neuronal loss [165], while P25 transgenic model underwent extensive non-apoptotic neuronal death [145]. Thus, from these perspectives, a direct comparison of these animal models would be challenging. Future experiments aimed to compare the efficacy of these models in recapitulating AD amyloid and tau pathologies would be illuminating.

Finally, models reviewed in this article were those without underlying genetic manipulations related to familial AD. There are potential models that their effects on neuroinflammation have not yet been evaluated on wild-type animals. For example, degeneration of the locus ceruleus through alkylating agent N-(2-chloroethyl)-N-ethyl-bromo-benzylamine (DSP4) in APP transgenic animals displayed increased neuroinflammation and exacerbation of plaque pathology and behavioral deficits [166]. Future studies on aged wild-type animals can provide evidence for application of this model in sporadic AD.

## Conclusion

Disease-specific animal models are indispensable for the understanding of possible disease mechanisms as well as for preclinical drug development. Undoubtedly, conventional transgenic models of AD are the basis of our today’s in-depth understanding of several mechanisms that are probably involved in AD. However, since all potential Alzheimer’s disease-modifying agents tested in these models have failed in phase-3 clinical trials, their application in drug discovery is under question.

Different strategies can be considered to bridge the gap between human AD pathology and rodent AD models. On one hand, major efforts should be undertaken to thoroughly characterize conventional animal models with newly available methods [167], to allow for more realistic translation of the results from animal models to human LOAD. On the other hand, etiology-based models should be established for LOAD [1]. Thus far, several hypotheses regarding the probable etiology of AD have been suggested, however, appropriate *in vivo* models to test these hypotheses are still lacking. In this review, we synthesized the current information about rodent models potentially compatible with the inflammation hypothesis of AD [13]. All in all, the choice of an animal model should be an informed decision on behalf of the investigator. Nevertheless, using etiology-based models of LOAD may create a breakthrough in understanding of the disease pathology, designing precise diagnostic modalities and discovery of effective therapeutic agents.

## Abbreviations

APP: amyloid precursor protein
Aβ: amyloid-β
CDK: cyclin-dependent kinases
IFN: interferon
hpTau: hyperphosphorylation of tau
IL: interleukin
ICV: intracerebroventricular
LOAD: late-onset Alzheimer’s disease
LPS: lipopolysaccharide
MHC: major histocompatibility complex
NGF: nerve growth factor
NFT: neurofibrillary tangles
OKA: okadaic acid
PHF: paired helical filaments
PolyI:C: polyriboinosinic-polyribocytidilic acid
PS1: presenilin-1
PP2A: protein phosphatase 2A
STZ: streptozotocin
TLR-4: toll-like receptor-4
TGF-β: transforming growth factor-β
TNF-α: tumor necrosis factor-alpha
